# Extended ECG Improves Classification of Paroxysmal and Persistent Atrial Fibrillation Based on P- and f-Waves

**DOI:** 10.3389/fphys.2022.779826

**Published:** 2022-03-04

**Authors:** Matthias Daniel Zink, Rita Laureanti, Ben J. M. Hermans, Laurent Pison, Sander Verheule, Suzanne Philippens, Nikki Pluymaekers, Mindy Vroomen, Astrid Hermans, Arne van Hunnik, Harry J. G. M. Crijns, Kevin Vernooy, Dominik Linz, Luca Mainardi, Angelo Auricchio, Stef Zeemering, Ulrich Schotten

**Affiliations:** ^1^RWTH University Hospital Aachen, Internal Medicine I, Cardiology and Vascular Medicine, Aachen, Germany; ^2^Cardiovascular Research Institute Maastricht (CARIM), Physiology, Maastricht, Netherlands; ^3^Department of Electronics, Information and Bioengineering, Politecnico di Milano, Milan, Italy; ^4^Center for Computational Modeling in Cardiology, Lugano, Switzerland; ^5^Department of Cardiology, Cardiovascular Research Institute Maastricht (CARIM), Maastricht University Medical Center, Maastricht, Netherlands; ^6^Ziekenhuis Oost Limburg, Genk, Belgium; ^7^Department of Cardiology, Radboud University Medical Center, Nijmegen, Netherlands; ^8^Instituto Cardiocentro Ticino, Lugano, Switzerland

**Keywords:** atrial fibrillation, paroxysmal, persistent, P-wave, f-wave, signal-averaged P-wave, P-wave variability, QRST-canceled ECG

## Abstract

**Background:**

The standard 12-lead ECG has been shown to be of value in characterizing atrial conduction properties. The added value of extended ECG recordings (longer recordings from more sites) has not been systematically explored yet.

**Objective:**

The aim of this study is to employ an extended ECG to identify characteristics of atrial electrical activity related to paroxysmal vs. persistent atrial fibrillation (AF).

**Methods:**

In 247 participants scheduled for AF ablation, an extended ECG was recorded (12 standard plus 3 additional leads, 5 min recording, no filtering). For patients presenting in sinus rhythm (SR), the signal-averaged P-wave and the spatiotemporal P-wave variability was analyzed. For patients presenting in AF, f-wave properties in the QRST (the amplitude complex of the ventricular electrical activity: Q-, R-, S-, and T-wave)-canceled ECG were determined.

**Results:**

Significant differences between paroxysmal (*N* = 152) and persistent patients with AF (*N* = 95) were found in several P-wave and f-wave parameters, including parameters that can only be calculated from an extended ECG. Furthermore, a moderate, but significant correlation was found between echocardiographic parameters and P-wave and f-wave parameters. There was a moderate correlation of left atrial (LA) diameter with P-wave energy duration (*r* = 0.317, *p* < 0.001) and f-wave amplitude in lead A3 (*r* = −0.389, *p* = 0.002). The AF-type classification performance significantly improved when parameters calculated from the extended ECG were taken into account [area under the curve (AUC) = 0.58, interquartile range (IQR) 0.50–0.64 for standard ECG parameters only vs. AUC = 0.76, IQR 0.70–0.80 for extended ECG parameters, *p* < 0.001].

**Conclusion:**

The P- and f-wave analysis of extended ECG configurations identified specific ECG features allowing improved classification of paroxysmal vs. persistent AF. The extended ECG significantly improved AF-type classification in our analyzed data as compared to a standard 10-s 12-lead ECG. Whether this can result in a better clinical AF type classification warrants further prospective study.

## Introduction

Atrial fibrillation (AF) and structural heart disease can lead to atrial structural remodeling, which is characterized by atrial dilatation (Osranek et al., 2005; Potpara et al., 2013), fibrosis (Marrouche et al., 2014), and fatty infiltrations (Haemers et al., 2017), all of which contribute to local conduction heterogeneities during sinus rhythm (SR) and AF. Shortening and increased heterogeneity of refractory periods (Frustaci, 1997; Nattel, 1999; Heijman et al., 2016; Opacic et al., 2016) occur as a consequence of ion channel remodeling. These structural and electrical changes enhance stability and progression of AF and reduce responsiveness to therapy (Schotten et al., 2001; Nguyen et al., 2009).

The standard 12-lead ECG is the gold standard to diagnose AF at the time of recording (Kirchhof et al., 2016) but also is increasingly used to characterize atrial conduction properties during SR and AF (Platonov, 2012; Lankveld et al., 2014, 2016; Potse et al., 2016). A routine 12-lead ECG is heavily filtered in order to visualize ECGs free of artifacts, leading to a loss of subtle, detailed information on electrical activation (Potse et al., 2016). Reported analyses of unfiltered 12-lead ECGs to stratify patients with AF are promising but limited by small cohort size (Fukunami et al., 1991; Dilaveris et al., 1998; Nault et al., 2009; Sasaki et al., 2015). Reports from larger cohorts, commonly based on retrospective datasets, are limited by the use of a low number of leads and set of parameters, filtering, and the short duration of the ECGs (Magnani et al., 2015; Nielsen et al., 2015).

The aims of this investigation were (I) to characterize P- and f-waves of patients with a history of AF, using an extended ECG (5 min recording and additional ECG leads), (II) to relate these characteristics to the clinically diagnosed AF type and (III) to study the added predictive value of the extended ECG compared to a standard 10-s 12-lead ECGs.

## Materials and Methods

### Study Population

All subjects were scheduled for AF ablation and enrolled in the AF ABlation (AFAB) registry. The study design was approved by the institutional review board (IRB: 16-4-208; NCT03075930) and conducted in accordance with the declaration of Helsinki and the International Conference on Harmonization Good Clinical Practice Guidelines. The patients were stratified according to the rhythm present during ECG recording (AF or SR). s We obtained informed consent in 279 consecutive patients, eligible for participation between January 2017 and August 2018 at Maastricht University Medical Center (MUMC +). Inclusion criteria were: documented AF, ECG recording performed with a (CAM-USB) (GE Healthcare, Eindhoven, The Netherlands) or YRS-100 (YourRhythmics BV, Maastricht, The Netherlands) device, scheduled for AF ablation, ≥ 18 years of age, and able and willing to give informed consent. Exclusion criteria were: emergency ablation and critical condition before ablation. Patients with heart rhythm other than SR or AF were excluded from analysis. With these inclusion and exclusion criteria, 247 (89%) out of 279 participants were included for this analysis. All participants were classified by their clinical type of AF, following the definition of the guidelines of the European society of cardiology (Hindricks et al., 2020):

• Paroxysmal: “AF that terminates spontaneously or with intervention within 7 days of onset”.
• Persistent: “AF that is continuously sustained beyond 7 days, including episodes terminated by cardioversion (drugs or electrical cardioversion) after ≥ 7 days”.

### ECG Recordings

The standard 10-s 12-lead ECGs were recorded at hospital admission. Subsequently, extended ECGs of at least 5 min lengths were recorded using the CAM-USB or YRS-100 ECG device. For SR and AF analysis, the extended ECG analysis sampling frequency was 500 Hz (CAM-USB Device) or 2,000 Hz (YRS-100 device), respectively.

**FIGURE 1:**
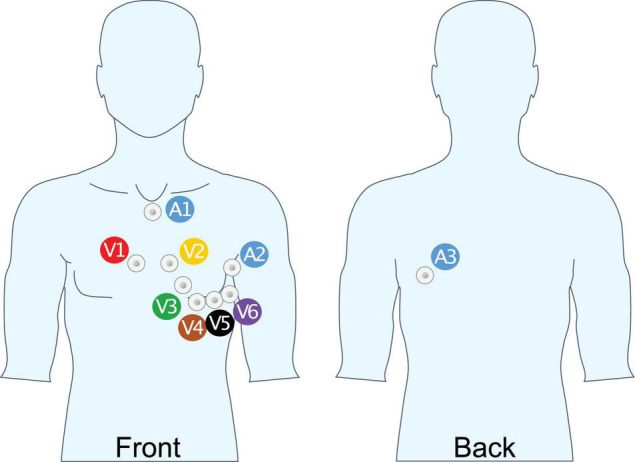
Unipolar lead placement for extended ECG recording. A1: Cranial end of sternum, beneath jugulum; A2: 8 cm above V6 mid-axillary line; A3: Same height as A2, above V9, 4 cm medial of posterior axillary line.

Apart from a longer recording, three additional leads were recorded with the extended ECGs. In previous work (Meo et al., 2013; Lankveld et al., 2014), these three additional lead locations, (A1, A2, and A3; Figure 1), were identified as locations with the highest sensitivity for P-wave complexity in persistent AF.

### ECG Analyses

#### Routine ECG Parameter Analysis

The P-wave duration, PQ, QRS, QT, and RR intervals were determined from the standard 10-s 12-lead ECGs using Yourrhythmics’ analysis software, based on the commercially available EN ISO 13485 certified Cardiolund ECG bibliotheque (Cardiolund AB 2018, Lund, Sweden)^1^ and checked by a cardiologist.

#### Extended ECG Parameter Analysis

All extended ECGs (both in SR and AF) were analyzed using custom-made software in MATLAB (2020b, The MathWorks, Natick, MA, United States). For signal-averaged P-wave parameter analysis of ECGs in SR, a baseline correction and 50 Hz notch filter to reduce power line interference were applied. Before f-wave analysis, the ECGs were filtered with a 1–100 Hz band-pass filter (3rd order Chebyshev, 20dB stop-band attenuation). A 50 Hz notch filter was applied in case of strong powerline interference.

Beside the routine ECG parameter analysis, there were three main ECG analysis calculated (for participants measured in SR: signal-averaged P-wave and the spatiotemporal P-wave variability analysis and for participants measured in AF: QRST-canceled f-wave analysis).

##### Signal-Averaged P-Wave Analysis

**FIGURE 2:**
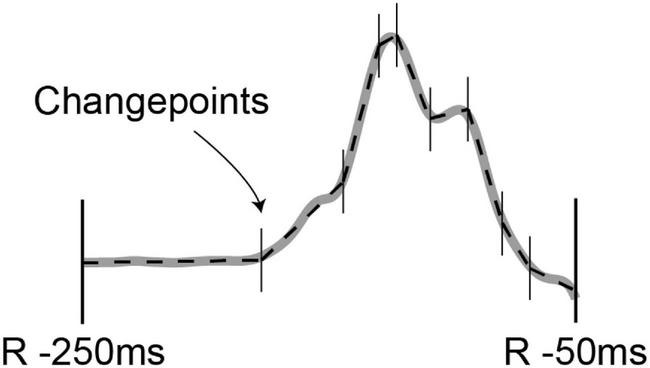
Averaged P-wave reconstruction by linear fits using MATLAB’s findchangepts algorithm.

To calculate a signal-averaged P-wave, the R-peaks were used for a first gross alignment of the P-waves. To correct for possible small PQ-interval changes, a window that contains the P-waves (e.g., R-peak −250 ms to R-peak −50 ms) was thereafter aligned until the maximal correlation between P-waves was achieved. This procedure takes all ECG leads and the entire signal within this window into account and is therefore independent of the shape of the P-wave. After signal-averaged P-waves were calculated for each lead, a global P-wave start and end were determined using a custom-made algorithm. To determine the P-wave start and end, the P-waves were filtered using a 0.5–75 Hz band-pass Butterworth filter. Then abrupt changes in the averaged P-waves were found using MATLAB’s function “*findchangepts*” (part of the signal toolbox) with the “*linear statistic*” option (Lavielle, 2005; Killick et al., 2012). In short, “*findchangepts”* detected abrupt changes (called changepoints) in mean and slope of a signal by fitting a linear line through segments of the signal (see Figure 2). The function added segments (and thus changepoints) as long as adding a segment reduces the total residual error by a predefined threshold (40 μV in our detection). The first and last linear fits with a slope ≥ 150 μV/s were used as the P-wave start and end of that particular ECG lead. The global P-wave start and end were then defined as the 10th percentile of the lead-specific P-wave start and the 90th percentile of all lead-specific P-wave end. These global P-wave start and end locations are used to calculate the parameters of the signal-averaged P-wave. For this project, leadwise calculation of P-wave area, amplitude, the terminal force in V1 (area from zero-crossing until global P-wave end), Shannon entropy (signal uncertainty), sample entropy (signal irregularity), and P-wave complexity (number of significant peaks in the averaged P-wave) were calculated using a custom-made software.

Signal-averaged P-wave parameter:

• Global P-wave duration: Start to end of the signal-averaged global P-wave measured in milliseconds.
• Area: Enclosed signal area between the start and end of the P-wave, measured in millivolts × milliseconds (Van Beeumen et al., 2010).
• Amplitude: Maximum to minimum peak measured in millivolt (Park et al., 2016).
• Terminal force: Area starting from zero-crossing of the biphasic P-wave in V1 to negative until the end of the P-wave, measured in millivolts and milliseconds (Magnani et al., 2015).
• Shannon entropy: A measure of data consumption to describe a signal. Shannon entropy measures the uncertainty and irregularity within the signal. A higher number reflects a higher uncertainty:

$$S\,h\,a\,n\,n\,o\,n\,e\,n\,t\,r\,o\,p\,y=-\sum_{i=1}^{10}P\,\left(x_{i}\right)\,l\,o\,g_{2}\,\left(P\,\left(x_{i}\right)\right)$$

where the voltage values of the P-wave were binned into *10* voltage bins *x*_*i*_, and *P(x_*i*_)* denotes the estimated probability of observing a voltage in bin *x*_*i*_. *x*_*i*_.
• Sample entropy: Estimate of the predictability and regularity within the signal. Higher values correspond to a higher irregularity of the signal (Alcaraz and Rieta, 2010):

$$S\,a\,m\,p\,l\,e\,e\,n\,t\,r\,o\,p\,y\,\left(m,r\right)=-l\,o\,g\,\frac{A^{m}\,\left(r\right)}{B^{m}\,\left(r\right)}$$

where *B^m^*(*r*) denotes the average number of matches of over all segments of length *m* in the P-wave within a threshold r, and *A^m^*(*r*) denotes the average number of matches of over all patterns of length *m* + *1*. In our study, we computed sample entropy using common values *m* = *2*, and r = *0.35* on signal averaged P-waves downsampled to 200 Hz.
• Complexity (Potse et al., 2016): The number of peaks and valleys in the P-wave morphology. A peak or valley was considered significant if its amplitude difference with adjacent valleys or peaks exceeded 10% of the average P-wave amplitude observed in that lead in all patients.

##### Spatiotemporal P-Wave Variability Analysis

The spatiotemporal P-wave variability was assessed on all ECG leads after 0.5–80 Hz band-pass and 50 Hz notch filtering. Besides the ECG leads, the first three principal components (PC1, PC2, and PC3) of the ECG leads (representing the three most representative directions of cardiac depolarization) were added to the analysis. The Principal Component Analysis is a statistical procedure that generates a set of uncorrelated and orthogonal variables from the original dataset (It, 2002). The spatiotemporal variability was analyzed by temporal, spatial, and morphological parameters such as the Euclidean distance, similarity index, and spatial similarity:

• Euclidean distance: For each lead and principal component, the median value of the N-1 euclidean distances (ED_n_) between each pair of consecutive P-waves (Laureanti et al., 2020a):

$$E\,D_{n}=\frac{\sqrt{\sum_{k=1}^{K}{\left(P_{n+1}\,\left[k\right]-P_{n}\,\left[k\right]\right)}^{2}}}{\sqrt{\sum_{k=1}^{K}{\left(P_{n+1}\,\left[k\right]\right)}^{2}}},n=1,2,\ldots ,N-1$$

where P_n_ is the vector of the n-th P-wave, k is the k-th sample of the wave, and N the total number of P-waves in the recording.
• Similarity index: For each lead and principal component, the median value of the N-1 cosines of the angle (Si_n_) between each pair of consecutive P-waves (Laureanti et al., 2020):

$$S\,I_{n}=\frac{{\text{P}}_{n}\cdot {\text{P}}_{n+1}}{||{\text{P}}_{n}||\cdot ||{\text{P}}_{n+1}||},n=1,2,\ldots ,N-1$$

where P_n_ is the n-th P-wave and N is the total number of P-waves in the recording.
• Spatial similarity: Total percentage of variance explained by the 2 first principal components computing applying PCA on each beat. A median value of all the beats was computed (Di Marco et al., 2012; Meo et al., 2018; Laureanti et al., 2020b).

Other sources of ECG-related variability (heart rate, heart axis, and noise level) were also measured as confounding factors, as described earlier (Laureanti et al., 2020b):

• Root Mean Square of Successive Difference (RMSSD) of the RR series.
• RMSSD of the heart axis series.
• Noise level: Median value of the SD of each isoelectric segment of 50 ms preceding each P-wave.

##### QRST-Canceled f-Wave Analysis

For ECGs recorded in AF, an adaptive singular value QRST-cancelation was applied (Lankveld et al., 2016). The QRST windows were aligned and clustered using hierarchical clustering with a minimal window correlation of 0.75. Average beat subtraction was performed for each QRST cluster separately using singular value decomposition to determine the average beat. The QRST windows forming a single cluster were blanked. The remaining signal consisted mainly of atrial AF activity reflected by f-waves, and it was analyzed for dimensional, frequency domain, and time domain parameters (dominant frequency, organization index, regularity index, spectral entropy, and f-wave amplitude) (Zeemering et al., 2018).

f-wave parameter:

• Dominant frequency: Frequency corresponding to the largest peak in the analyzed power spectrum (Zeemering et al., 2018).
• Organization index: Ratio of the cumulative areas of two peaks with the strongest power in the power spectrum to the area of the entire power spectrum (Uldry et al., 2012).
• Regularity index: The relative contribution of the dominant frequency and its harmonic frequencies to the entire power spectrum (Dibs et al., 2008).
• Spectral entropy: A measure of uniformity of the spectral content (Uldry et al., 2012).
• f-wave amplitude: Mean amplitude of the f-waves (Nault et al., 2009).

#### Classification of Atrial Fibrillation Type

The subjects were classified as paroxysmal or persistent AF using a lasso logistic regression approach. Classification performance was assessed by cross-validated area under the curve (AUC) of the receiver-operating characteristics (ROC). Cross-validation was performed with 5 folds and 20 repetitions. To investigate the added value of the extended ECG with respect to a standard 10-s 12-lead ECG, the classification performance was compared between models containing only parameters that are (or can be) determined from a standard ECG and models containing parameters that can only be determined from an extended ECG.

For ECGs in SR, the models with the following input variables were used: (I) only standard ECG parameters (see 12-lead ECG parameters in Supplementary Table 1); (II) standard ECG parameters + clinical characteristics as shown in Table 1; (III) all ECG parameters including the parameters that can only be determined from an extended ECG (all parameters in Supplementary Table 1); and (IV) all ECG parameters + clinical characteristics.

**TABLE 1:** Baseline characteristics.

		Total	Paroxysmal	Persistent	*P*
*N* =		247	151	96	
Male		66% (162)	60% (90)	75% (72)	0.014
Age	[years]	63.9 ± 8.7	63.5 ± 8.8	64.5 ± 8.7	0.413
Height	[cm]	176 ± 9	176 ± 10	176 ± 9	0.530
Weight	[kg]	86.5 ± 16.8	83.5 ± 14.3	91.3 ± 19.1	0.003
BMI	[kg/m^2^]	27.9 ± 4.8	27.1 ± 4.2	29.3 ± 5.4	<0.001
CHA_2_DS_2_-VASc		2.0 ± 1.5	2.0 ± 1.6	2.1 ± 1.4	0.219*
Symptomatic heart failure		15% (36)	6% (9)	30% (27)	<0.001
Hypertension		47% (116)	46% (70)	52% (50)	0.896
Age 65–74 years		42% (103)	43% (65)	38% (40)	0.600
Age > = 75 years		12% (29)	10% (15)	15% (14)	0.312
Diabetes mellitus		8% (20)	7% (11)	9% (9)	0.634
Stroke		10% (21)	11% (14)	8% (7)	0.643
Vascular disease		18% (41)	15% (21)	23% (20)	0.155
**Echocardiographic findings**					
LVEF	[%]	57 ± 8	59 ± 6	53 ± 10	<0.001*
LAdiameter	[mm]	43 ± 6	42 ± 5	44 ± 6	0.084
LA volume	[mL]	86 ± 26	80 ± 22	95 ± 28	0.001*
RA volume	[mL]	63 ± 28	56 ± 23	72 ± 31	<0.001*
**AF Baseline characteristics**					
AF known for	[months]	59 ± 66	54 ± 66	68 ± 65	0.014*
First ablation		74% (183)	87% (120)	82% (63)	0.301
Antiarrhythmic drug treatment					
Flecainide		23% (57)	30% (46)	12% (11)	0.002
Betablocker		39% (97)	36% (54)	45% (43)	0.021
Sotalol		22% (54)	25% (37)	18% (17)	0.513
Amiodarone		9% (22)	4% (6)	17% (16)	<0.001
Digoxine		10% (25)	7% (11)	15% (14)	0.028
Anticoagulation drug treatment		97% (230)	96% (141)	99% (89)	0.258

Values are given as mean ± SD or percentage (number). P-values were calculated with Fisher’s exact test or independent t-test, unless indicated other. *P-value was calculated with Mann-Whitney U-test. BMI, Body mass index; LVEF, Left ventricular ejection fraction; Anticoagulation, Either Vitamin-K antagonist or non-vitamin K antagonist anticoagulation.

For ECGs in AF, the following input variable sets were used: (I) all f-wave parameters that can be calculated from a standard 10-s 12-lead ECG (i.e., Dominant Frequency, organization index, regularity index, spectral entropy, and f-wave amplitude); (II) f-wave parameters from a standard ECG + clinical characteristics; (III) all f-wave parameters including the parameters that can only be determined from an extended ECG (all parameters in Supplementary Table 2); and (IV) all f-wave parameters + clinical characteristics.

#### Statistical Methods

Statistical analyses were performed using IBM statistical package for the social sciences (SPSS) (IBM Corp., 2019, Version 26.0. Armonk, NY, United States), R (version 3.5.1, 2018, The R Foundation for Statistical Computing; Package for lasso regression: “glmnet”; Friedman et al., 2010), and MATLAB. Continuous variables were tested for normality using the Kolmogorov-Smirnov test and reported as mean ± *SD*. The comparison of two groups were calculated using a Mann-Whitney *U* or Independent *t*-test. For the spatiotemporal P-wave variability, a generalized linear regression model was built for each spatiotemporal P-wave variability parameter and adjusted for all sources of ECG-related variability to assess if a significant association was present. In the generalized linear model the AF class was the dependent variable, while the spatiotemporal P-wave variability parameters and the confounding factors (i.e. the other sources of ECG-related variability such as RMSSD of the RR series, RMSSD of the heart axis, Noise Level) were the independent variables. The categorical variables were tested with Fishers exact test and reported in number and percentages. The Pearson’s correlation coefficient was computed between clinical and ECG parameters. The classification performances of the different models were assessed using a paired samples *t*-test on the cross-validated AUC (please find the detailed description in paragraph “Classification of AF type”). A *P*-value of 0.05 was considered statistically significant.

## Results

### Study Population

Out of 247 participants included in this study, 164 (66%) were in SR and 83 (33%) were in AF during the ECG recording. The majority of patients in SR were paroxysmal patients with AF (121 paroxysmal vs. 43 persistent) while the majority of patients in AF were persistent patients with AF (31 paroxysmal vs. 52 persistent).

### Baseline Characteristics

The persistent patients with AF were more often male, were heavier, had a higher body mass index, and suffered more often from symptomatic heart failure, whereas there was no difference in CHA_2_DS_2_VASc score between groups (Table 1, *P* = 0.219). The time since first AF diagnosis was longer in persistent AF, and echocardiography revealed lower left ventricular ejection fractions and larger right and left atria. This reflects a higher degree of atrial remodeling in patients with persistent AF. Anticoagulation usage was not different between groups, and anti-arrhythmic drugs were more often prescribed in the persistent AF group.

### Univariate Differences in P-Wave and f-Wave Features Between Patients With Paroxysmal and Persistent Atrial Fibrillation

In standard ECGs, no differences in P-wave duration (118 ± 16 ms vs. 113 ± 18 ms, *p* = 0.06) or PQ-interval (180 ± 28 ms vs. 184 ± 27 ms, *P* = 0.271) were observed between paroxysmal and persistent AF, respectively (Supplementary Table 1).

In contrast, in extended ECG, significant differences between groups were observed in the analysis of P- and f-waves. Table 2 shows all parameters with significant differences between paroxysmal and persistent AF, a complete list of parameters is shown in Supplementary Table 1. For signal-averaged P-waves in patients with paroxysmal compared to persistent AF showed both lower values (for P-wave area lead V2, Shannon entropy lead aVL, Sample entropy lead A2, I, and aVR) and higher values (for P-wave amplitude lead A2 and I Shannon entropy lead V1 and aVF Sample entropy V1 and V2) depending on the investigated lead. In contrast, the complexity was lower for any analyzed lead (A1, II, V1, V5, V6, aVR) in paroxysmal compared to persistent AF. The spatiotemporal P-wave variability analysis resulted in both lower temporal (higher Euclidean distance and lower similarity index) as well as lower spatial similarity in persistent AF in several leads (Table 2). In f-wave analyses, dominant frequency was higher in A1 and f-wave amplitude was lower in aVF, V4, V5, V6, and A3 for persistent AF (Table 3). Other f-waves parameters showed only subtle differences between groups (Supplementary Table 2).

**TABLE 2:** P-wave parameters with differences for comparison between patients with paroxysmal and persistent AF.

		Total	Paroxysmal	Persistent	*P*
*N* =		164	121	43	
**Signal-averaged P-wave**					
Area [mV*ms]	V2	3.62 ± 4.19	3.19 ± 1.33	4.82 ± 7.82	0.024*
Amplitude [mV]	A2	0.058 ± 0.03	0.058 ± 0.019	0.057 ± 0.05	0.037*
	I	0.087 ± 0.043	0.089 ± 0.033	0.083 ± 0.065	0.029*
Shannon entropy [au]	V1	3.03 ± 0.16	3.04 ± 0.13	2.97 ± 0.23	0.033*
	aVF	3.11 ± 0.11	3.12 ± 0.11	3.08 ± 0.12	0.01*
	aVL	3.01 ± 0.13	2.99 ± 0.14	3.05 ± 0.12	0.033*
Sample entropy [au]	A2	0.231 ± 0.078	0.225 ± 0.078	0.249 ± 0.076	0.035*
	I	0.225 ± 0.089	0.213 ± 0.072	0.259 ± 0.119	0.006*
	V1	0.221 ± 0.044	0.225 ± 0.042	0.211 ± 0.046	0.024*
	V2	0.243 ± 0.087	0.253 ± 0.092	0.215 ± 0.065	0.009*
	aVR	0.218 ± 0.055	0.212 ± 0.051	0.237 ± 0.063	0.011*
Complexity [N]	A1	2.7 ± 1.7	2.4 ± 1.5	3.3 ± 2.1	0.008*
	II	2 ± 1.3	1.9 ± 1.2	2.4 ± 1.6	0.025*
	V1	2.2 ± 0.9	2.1 ± 0.7	2.4 ± 1.4	0.037*
	V5	2.1 ± 1.3	2 ± 1.2	2.6 ± 1.6	0.024*
	V6	2 ± 1.3	1.8 ± 1.1	2.4 ± 1.7	0.009*
	aVR	1.9 ± 1.3	1.7 ± 1	2.4 ± 1.9	0.014*
**Spatiotemporal P-wave variability**					
Euclidean Distance	A1	(4.10 ± 2.10) × 10^–1^	(3.91 ± 2.03) × 10^–1^	(4.67 ± 2.21) × 10^–1^	0.015^§^
	A2	(4.71 ± 2.22) × 10^–1^	(4.47 ± 2.06) × 10^–1^	(5.40 ± 2.55) × 10^–1^	0.001^§^
	I	(4.83 ± 2.63) × 10^–1^	(4.52 ± 2.41) × 10^–1^	(5.73 ± 3.07) × 10^–1^	0.001^§^
	II	(2.99 ± 1.32) × 10^–1^	(2.86 ± 1.18) × 10^–1^	(3.37 ± 1.63) × 10^–1^	0.009^§^
	V6	(4.11 ± 1.71) × 10^–1^	(3.87 ± 1.48) × 10^–1^	(4.80 ± 2.12) × 10^–1^	0.002^§^
	aVR	(3.30 ± 1.64) × 10^–1^	(3.09 ± 1.44) × 10^–1^	(3.89 ± 2.03) × 10^–1^	0.001^§^
	PC1	(2.37 ± 1.14) × 10^–1^	(2.28 ± 1.09) × 10^–1^	(2.62 ± 1.26) × 10^–1^	0.009^§^
Similarity Index	A1	(9.08 ± 0.93) × 10^–1^	(9.19 ± 0.81) × 10^–1^	(8.76 ± 1.16) × 10^–1^	0.011^§^
	A2	(8.82 ± 1.20) × 10^–1^	(9.01 ± 0.89) × 10^–1^	(8.28 ± 1.71) × 10^–1^	< 0.001^§^
	I	(8.63 ± 1.48) × 10^–1^	(8.86 ± 1.20) × 10^–1^	(7.96 ± 1.96) × 10^–1^	0.001^§^
	II	(9.57 ± 0.35) × 10^–1^	(9.61 ± 0.30) × 10^–1^	(9.45 ± 0.45) × 10^–1^	0.008^§^
	V6	(9.18 ± 0.64) × 10^–1^	(9.29 ± 0.49) × 10^–1^	(8.87 ± 0.89) × 10^–1^	0.001^§^
	aVR	(9.44 ± 0.55) × 10^–1^	(9.52 ± 0.45) × 10^–1^	(9.21 ± 0.72) × 10^–1^	0.002^§^
	PC1	(9.72 ± 0.27) × 10^–1^	(9.76 ± 0.22) × 10^–1^	(9.61 ± 0.37) × 10^–1^	0.001^§^
Spatial similarity [%]		94.40 ± 2.70	94.75 ± 2.30	93.33 ± 3.46	0.001^§^

Values are given as mean ± SD. *P-value calculated with Mann-Whitney U-test.^§^ P-value calculated using a generalized linear regression model adjusted for other ECG-related variability.

**TABLE 3:** Differences for f-wave parameters in comparison of patients with paroxysmal and persistent AF.

		Total	Paroxysmal	Persistent	*P*
*N* =		83	31	52	
Dominant frequency [Hz]	A1	6.50 ± 0.91	6.23 ± 0.73	6.65 ± 0.98	0.047*
f-wave amplitude [μV]	A3	49.82 ± 16.33	56.18 ± 18.88	46.22 ± 13.61	0.011*
	V4	67.98 ± 21.20	74.22 ± 21.55	64.56 ± 20.40	0.036*
	V5	59.15 ± 18.04	64.48 ± 18.07	56.14 ± 17.48	0.03*
	V6	52.39 ± 15.37	57.79 ± 16.97	49.34 ± 13.62	0.014*
	aVF	83.05 ± 28.47	91.99 ± 31.73	77.99 ± 25.38	0.043*

Values are given as mean ± SD. *P-value was calculated with Mann-Whitney U-test. Values are given as mean ± SD. *P-value was calculated with Mann-Whitney U-test.

### Classification of Atrial Fibrillation Type

**FIGURE 3:**
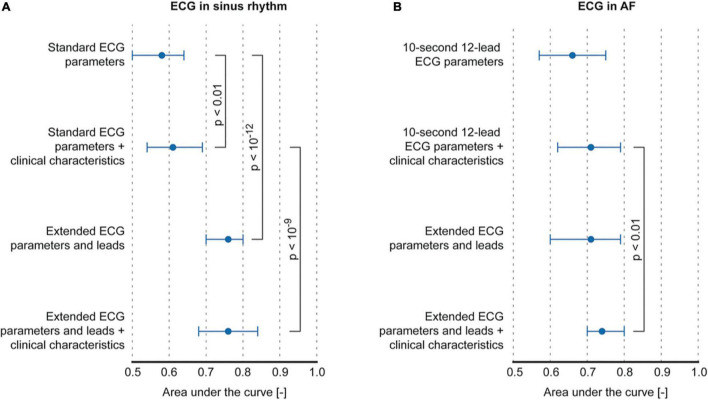
Classification performance of models using P-wave or f-wave features. **(A)** Comparison of classification performance between standard ECG parameters and extended ECG P-wave parameters and leads, with and without controlling for clinical characteristics. **(B)** Comparison between classification performance of f-wave parameters that can be computed on a 10-s 12-lead ECG vs. parameters that can be computed on the extended ECG.

The classification of AF in paroxysmal and persistent AF was poor based on P-wave parameters that could be determined from a standard ECG (maximum AUC: 0.58, interquartile range (IQR) 0.50–0.64 using P-wave duration) and improved only marginally by including clinical characteristics (AUC: 0.61, IQR 0.54–0.69, *p* < 0.01 using P-wave duration and age, sex, weight, BMI, and heart failure). The classification based on extended ECG leads and parameters improved performance to an AUC of 0.76, IQR 0.70–0.80 (*p* < 0.001 compared to standard ECG parameters using amplitude I, Shannon entropy I and aVL, Sample entropy V2, complexity III and aVR, and similarity index V6). Controlling for clinical characteristics did not alter classification performance of the extended ECG (*r* = 0.76, IQR 0.68–0.84, Figure 3 and Table 4).

**TABLE 4:** Classification of AF type based on P-wave parameters and f-wave parameters.

Model	Parameters and leads	AUC	Sensitivity	Specificity
	***P-wave parameters (n* = *119 paroxysmal AF, 40 persistent AF)***			
Standard ECG parameters	P-wave duration	0.58 (0.50−0.64)	0.54 (0.48−0.65)	0.50 (0.38−0.72)
Standard ECG parameters with clinical characteristics	P-wave duration Age Sex Weight BMI Heart failure	0.61 (0.54−0.69)	0.71 (0.62−0.78)	0.50 (0.38−0.62)
Extended ECG	Amplitude I Entropy I and aVL Sample entropy V2 Complexity III and aVR Similarity index V6	0.76 (0.70−0.80)	0.68 (0.62−0.75)	0.62 (0.50−0.75)
Extended ECG with clinical characteristics	Area V2 Entropy A2, I and aVL Complexity III and V5 Similarity index A2, PC2 and I Weight BMI Heart failure	0.76 (0.68−0.84)	0.79 (0.74−0.83)	0.62 (0.50−0.75)
	***f-wave parameters (n* = *30 paroxysmal AF, 53 persistent AF)***			
10s 12-lead ECG parameters	f-wave Amplitude V6	0.66 (0.57−0.75)	0.50 (0.33−0.62)	0.73 (0.55−0.80)
10s 12-lead ECG parameters with clinical characteristics	f-wave Amplitude V6 Sex Weight BMI Heart failure	0.71 (0.62−0.79)	0.50 (0.50−0.67)	0.71 (0.64−0.80)
Extended ECG	f-wave Amplitude A3 Dominant frequency A1 f-wave amplitude Dispersion A1	0.71 (0.60−0.79)	0.67 (0.50−0.79)	0.73 (0.60−0.82)
Extended ECG with clinical characteristics	f-wave Amplitude A3 f-wave amplitude Dispersion A1 Sex BMI Heart failure	0.74 (0.70−0.80)	0.67 (0.50−0.67)	0.73 (0.60−0.80)

*Values are given as median and interquartile range.*

In patients with an ECG recorded during AF, f-wave amplitude in V6 (AUC 0.66, IQR 0.57–0.75) achieved maximum classification performance when considering only ECG parameters that could be computed on a standard ECG (Figure 3B and Table 4). No significant improvement was achieved by controlling for clinical characteristics (AUC 0.71, IQR 0.62–0.79, using sex, weight, BMI, and heart failure). f-wave parameters derived from the extended ECG showed similar performance (AUC 0.71, IQR 0.60–0.79) mainly using information from extended leads on f-wave amplitude (lead A3) and temporal dispersion of f-wave amplitude (lead A1). The best performing model was obtained by combining extended ECG parameters and leads, and clinical characteristics (AUC 0.74, IQR 0.70–0.80, *p* < 0.01, using amplitude A3, amplitude dispersion A1, sex, BMI, and heart failure compared to a model based on standard ECG parameters and clinical characteristics).

The AF classification improved if only ablation-naive patients were included in the analysis for both the multivariable logistic P-wave model combining extended ECG parameters and leads, and clinical characteristics (AUC 0.83, IQR 0.76–0.87, sensitivity 80%, specificity 68%) as well as for the f-wave model solely based on f-wave amplitude in lead V6 (AUC 0.80, IQR 0.72–0.93, sensitivity 85%, specificity 68%) (Supplementary Table 4).

### Correlation With Atrial Remodeling

**FIGURE 4:**
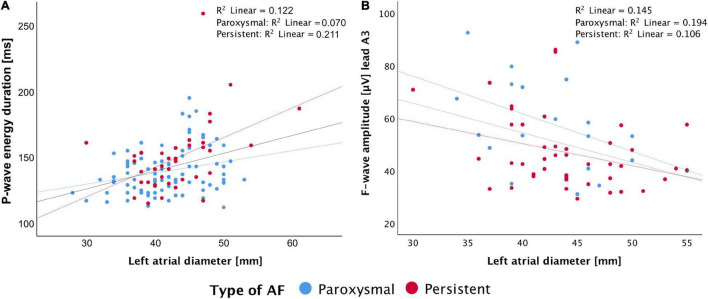
Correlation of left atrial diameter with P-wave energy duration **(A)** and f-wave amplitude in lead A3 **(B)**.

The correlation to clinical features was performed in subgroups with only the available data. We found significant correlations between P- and f-wave features and CHA_2_DS_2_-VASc score, LVEF, left atrial (LA) diameter, LA volume, and right atrial (RA) volume (for number of analyzed subgroup and best performing lead see Supplementary Table 3). The best correlation with LA diameter was found for the signal-averaged P-wave energy duration (all:*r^2^* = 0.317, *P* < 0.001; paroxysmal: *r^2^* = 0.249, *P* = 0.016; persistent: *r^2^* = 0.493, *P* = 0.002, *N* = 132, Figure 4A), similarity index in aVF (all: *r^2^* = −0.220, *P* = 0.016; paroxysmal: *r^2^* = −0.231, *P* = 0.032; persistent: *r^2^* = −0.130, *P* = 0.463, *N* = 123), and f-wave amplitude in A3 (all: *r^2^* = −0.389, *P* = 0.002; paroxysmal: *r^2^* = −0.486, *P* = 0.03; persistent: *r^2^* = −0.353, *P* = 0.02, *N* = 63, Figure 4B).

## Discussion

In our prospective investigation, the features from a signal-averaged P-wave and spatiotemporal variability in SR recordings as well as QRST-canceled f-wave analyses in AF recordings, all calculated from an extended ECG (5-min recording and additional ECG leads), revealed significant differences in ECGs of patients with paroxysmal and persistent AF, during both SR and AF. Furthermore, significant correlations were found between electrophysiological characteristics and echocardiographic measures of atrial remodeling. Finally, the AF-type classification performance was significantly improved by using parameters obtained from the extended ECG.

### Differences of P-Wave and f-Wave Features Between Patients With Paroxysmal and Persistent Atrial Fibrillation

In routine ECG parameter calculated from the standard 10-s 12-lead ECG, no significant differences between paroxysmal and persistent AF were found (Supplementary Table 1). In contrast, in extended ECG, the signal-averaged P-wave revealed significant differences in most leads and for nearly all parameters. The signal-averaged P-wave duration was significantly longer compared with the P-wave duration measured in standard ECGs. However, the signal-averaged P-wave duration showed a significant but weak correlation to left atrial dimensions (diameter and volume). More complex SR conduction patterns in more advanced stages of AF are likely to result in a higher P-wave complexity with more, but smaller changes in morphology. These changes will potentially be filtered out in routine ECGs and therefore remain undetected, as demonstrated by Potse et al. (2016). Routinely filtered P-waves may therefore appear shorter and potentially underestimate the duration of atrial electrical activity compared to signal-averaged P-wave. Although we did not perform any filtering of the raw ECG apart from baseline correction and 50 Hz noise suppression, the temporal alignment and averaging of hundreds of P-waves greatly suppresses the influence of noise in general. Furthermore, during P-wave alignment, the windows with deviating P-wave morphology compared to the running signal-averaged P-wave estimate were excluded (correlation coefficient < 0.9). In our data, P-waves in persistent AF calculated with routine ECG parameters showed a tendency to be shorter when measured using the Cardiolund package and longer when measured from the signal-averaged P-wave compared to paroxysmal AF cases (Table 2 and Supplementary Table 1). This is an effect potentially caused by routine filtering settings in standard ECG. Despite the higher resolution a signal-averaged P-wave provides, the analysis of routine dimensional parameters like amplitude and area showed only differences between the groups in a few leads. In contrast, the morphological analysis (Shannon and Sample entropy, complexity) of the signal-averaged P-wave revealed significant differences in all leads. More complex conduction patterns in persistent AF are therefore probably better reflected by morphological changes in signal-averaged P-waves than in single P-waves analyzed with routine ECG parameters.

In addition, the longer recording of our extended ECG allows analysis of variability and beat-to-beat changes in atrial depolarization pathways. Higher P-wave variability has been linked to higher risk of developing AF (Conte et al., 2017). Pezzuto et al. (2018) demonstrated that beat-to-beat P-wave variability is associated with the degree of heterogeneous conduction and the variability of the sinoatrial node’s exit point. According to these findings, we hypothesized a higher P-wave variability in persistent AF, proving a more compromised substrate with worsening of the pathology. This hypothesis is confirmed in our study. In a similar study by Laureanti et al. (2020a), no difference was found between the two types of AF inside the Swiss-AF cohort. However, a comparison between our population and the Swiss-AF cohort shows major demographic, treatment, and methodologic differences.

The extended ECGs recorded during AF show significant differences in dominant frequency and f-wave amplitude between paroxysmal and persistent AF (Table 3). These findings are in line with previous work in our group, in which frequency and f-wave amplitude were able to identify patients likely to benefit from catheter ablation, thereby identifying patients with a more complex AF substrate (Lankveld et al., 2016).

### Correlation With CHA_2_DS_2_-VASc Score and Echocardiographic Parameters

In subgroup analysis, a moderate correlation for P- and f-wave parameters in extended ECG with the CHA_2_DS_2_VASc score and echocardiographic parameters (Supplementary Table 3) were found. The extended ECG parameters (signal-averaged P-wave energy duration, spatiotemporal P-wave similarity index aVF, and QRST-canceled f-wave amplitude A3) showed most promising correlation with LA diameter. Furthermore, cardiovascular comorbidities (CHA_2_DS_2_VASc) correlated significantly with P-wave complexity I, Euclidian distance V4, and organization index I. This illustrates that the propagation of the electrical activity in structural remodeled and enlarged atria requires more time, and shows more complex propagation patterns for complete activation (Schotten et al., 2011; Haemers et al., 2017). The link between atrial structural and electrical remodeling is that in persistent patients with AF, the pathologic echocardiographic and ECG findings correlate better, due to more pronounced atrial remodeling.

### Added Value of the Extended ECG

The novelty of this report is that patients were not preselected in terms of heart rhythm, and analysis was performed across all available leads in a 12-lead ECG and even further extended on 3 additional leads in close proximity to the atria. Furthermore, the following 4 ways of ECG analysis were performed and compared (Potpara et al., 2013): routine ECG parameters (Osranek et al., 2005), signal-averaged ECG analysis (Marrouche et al., 2014), spatiotemporal P-wave analysis, and (Haemers et al., 2017) QRST-canceled f-wave analysis. The precordial leads encircle the longitudinal axis of the ventricles and provide a planar view of predominantly ventricular electrical activity. Therefore, in the extended ECG, we defined a more cranial plane to provide more insight in atrial electrical activity. In our cohort, significant differences were found in these atrial leads for several P- and f-wave parameters between paroxysmal and persistent patients with AF (Tables 2, 3). Furthermore, lead A1 and A3 correlated well with clinical findings of atrial remodeling in echocardiography. Apart from the additional atrial leads, the longer recording time of the extended ECG furthermore enables the calculation of more sophisticated parameters like spatiotemporal P-wave variability and f-wave dispersion. AF-type classification based on extended ECG leads and parameters significantly improved the performance with respect to the standard ECG parameters, indicating that the extended ECG leads and parameters contain additional information reflecting the electrophysiological properties of the atria (Table 4).

The measured values of traditional ECG parameters (Supplementary Table 1) show a wide variety, which may relate mainly to the process and inconsistent variety of atrial cardiomyopathy associated with structural changes (e.g., enlarged atria, or fibrosis) and, on the other hand with electrical changes with a higher degree of electrical activity disturbance. This complex combination of structural and functional changes seems, based on our data, not adequately represented by “traditional” ECG parameters like P-wave duration, amplitude, and area. As can be seen in existing evidence, both can be found, that is, longer (Bayés de Luna et al., 1988) and shorter (Nielsen et al., 2015) P-wave duration. In contrast, the extended ECG parameter like entropy, complexity, Euclidean distance, and similarity index show likewise tendencies toward one (higher or lower values) in persistent compared to paroxysmal types of AF across all leads. In our perspective, the complex morphological changes by different stages of atrial cardiomyopathy may be more pronounced in the extended ECG parameters.

The added value of our findings is that in extended ECG analysis, the patients with persistent AF measured in SR showed a longer global P-wave duration, lower amplitude (V2, aVR), and area (A2), combined with a higher complexity (A1, II, V1, V5, V6, aVR), suggesting slower and more irregular conduction properties compared to paroxysmal patients with AF in SR.

Ongoing AF in patients with persistent AF was characterized by a higher dominant frequency (A1) and lower f-wave amplitudes (A3, V4-V6, aVF) indicating shorter AF cycle lengths and smaller fibrillation waves compared to patients with paroxysmal AF.

Based on our data, there is no fixed lead and parameter combination performing best but left-oriented leads were included in all models, while right-oriented models were only included in the P-wave models. This suggests that during AF, there is especially an increased complexity in the left atrium in persistent AF, while during SR, a biatrial perspective is needed to capture these subtle differences in conduction characteristics.

However, the novel atrial lead positions and extended ECG parameter should not be considered as a replacement, but a complement to routine 12-lead ECG to support non-invasive diagnostics of atrial conduction properties.

### Future Applications

Most of the commercially available ECG devices offer the ability to add 3 lead positions to standard 12-lead ECG and allow recordings of long (5 min) and unfiltered ECGs. In particular, more advanced atrial remodeling was well-reflected by more pronounced spatiotemporal P-wave variability and complexity in signal-averaged P-waves and by more pronounced f-wave changes. Future applications of this technology may include staging of atrial remodeling potentially used in patient-tailored therapeutic decision-making. Because of higher AUC in the subgroup of ablation-naive patients, the upcoming studies should focus on first-ablation patients. To prove this hypothesis, further research in the context of prospective clinical trials is needed. Due to the extensive amount of data resulting from high resolution, multiple leads, and long recordings, the data are suitable for further analysis by artificial intelligence algorithms.

### Study Limitations

Although the investigated cohort represents one of the largest groups in which an extensive, detailed, and structured P- and f-wave analysis was performed so far and we found evidence for significant differences in between paroxysmal and persistent patients with AF, the findings were subtle in many cases. To ascertain the found evidence, it should be addressed by even larger cohorts in the upcoming prospective investigations. Echocardiographic data within the study cohort were available in 80% of all participants. We therefore excluded ultrasound data for the multivariate logistic regression models. We did not cardiovert or administer drugs before measurement and measured the patient’s ECG on admission independent of rhythm status at that moment. Because of differences in interindividual symptoms and limited reliability of self-described presence of AF, we cannot provide reliable data on how long the measured heart rhythm was present before measurement. The different sample frequencies of the used ECG devices (500/2,000 Hz) would neither affect the routine, spatiotemporal P-wave variability nor the QRST-canceled f-wave analysis. For signal-averaged P-waves, a higher sample frequency potentially adds information in the very high frequency range (250–1,000 Hz). However, this is not to be expected as the averaging procedure acts as a low-pass filter that was supported by the fact that we found no significant differences in terms of parameters computed on ECGs recorded with the two utilized devices.

## Conclusion

The extended ECG with novel atrial lead positions combined with prolonged recordings enabling signal-averaged P-wave, spatiotemporal P-wave variability, and QRST-canceled f-wave analysis allowed extraction of ECG features for classification of paroxysmal and persistent AF. Our parameters and lead combinations correlated with the echocardiographic findings of atrial remodeling and identified paroxysmal and persistent AF with moderate to good classification.

## Data Availability Statement

The raw data supporting the conclusions of this article will be made available by the authors upon reasonable request.

## Ethics Statement

The studies involving human participants were reviewed and approved by Ethic review board, University Maastricht. The patients/participants provided their written informed consent to participate in this study.

## Author Contributions

MZ developed the study concept and design, measured the participants, analyzed the P- and f-waves, interpreted the results, and wrote the first draft of the manuscript. RL developed the spatio-temporal variability analysis, interpreted the results, and wrote the sections of P-wave variability. BH developed the automated P-wave marking, measured the participants, and wrote the method sections of P-wave analysis. LP contributed to conception and design of the study. SP organized the database. NP and MV measured participants. AH developed the automated P-wave marking. SV, AHu, HC, KV, and DL contributed to conception, design, and interpretation of the results of the study. LM and AA developed the spatio-temporal variability analysis and interpreted the results. SZ performed statistical analysis, wrote the section of statistical analysis, contributed to the conception, design and interpretation of the results of the study. US developed the study concept and design, analyzed the P- and f-waves, and interpreted the results. All authors contributed to manuscript revision, read, and approved the submitted version.

## Conflict of Interest

AA was a consultant for Biosense Webster, Boston Scientific, Medtronic, and Microport CRM; Intellectual property: Biosense Webster, Boston Scientific, and Microport CRM; Speaker fees: Boston Scientific, Medtronic, Microport CRM, and Philips. US was shareholder of YourRhythmics BV and consultant for Roche, EP Solutions, and YourRhythmics BV. KV was a consultant for Biosense Webster, Medtronic, Philips, Abbott. The remaining authors declare that the research was conducted in the absence of any commercial or financial relationships that could be construed as a potential conflict of interest.

## Publisher’s Note

All claims expressed in this article are solely those of the authors and do not necessarily represent those of their affiliated organizations, or those of the publisher, the editors and the reviewers. Any product that may be evaluated in this article, or claim that may be made by its manufacturer, is not guaranteed or endorsed by the publisher.

## Footnotes

1
https://cardiolund.com/ecg-parser/

## Abbreviations

AF: Atrial fibrillation
AUC: Area under the curve
ECG: Electrocardiogram
IQR: Interquartile range
LA: Left atrium
RA: Right atrium
RMSSD: Root Mean Square of Successive Difference
ROC: Receiver-operating characteristics
SR: Sinus rhythm.
