# Supplementary material for: Extended ECG Improves Classification of Paroxysmal and Persistent Atrial Fibrillation Based on P- and f-Waves
Source: Front Physiol. 2022 Mar 4;13:779826. doi: 10.3389/fphys.2022.779826 (PMC8931504; doi:10.3389/fphys.2022.779826)
Supplement: Supplementary file 1 [file Data_Sheet_1.docx]

**Supplemental material**

**Extended ECG Improves Classification of Paroxysmal and Persistent Atrial Fibrillation based on P- and f-waves**

**ECG Characteristics of Paroxysmal and Persistent AF**

Matthias Daniel Zink*^1,2^, MD; Rita Laureanti*^3,4^ PhD; Ben JM Hermans*^2^, PhDLaurent Pison^2,5,6^, MD, PhD; Sander Verheule, PhD^2^; Suzanne Philippens^5^; Nikki Pluymaekers^2,5^, MD; Mindy Vroomen^2,5^, MD, PhD; Astrid Hermans^2,5^, MD; Arne van Hunnik^2^_,_ PhD; Harry J.G.M. Crijns^2,5^, MD, PhD; Kevin Vernooy^2,5,7^, MD, PhD; Dominik Linz, MD, PhD,^5^ Luca Mainardi, PhD^3^; Angelo Auricchio^4,8^, MD, PhD; PhD; Stef Zeemering^2^, PhD; Ulrich Schotten^2^, MD, PhD

*^1^RWTH University Hospital Aachen, Internal Medicine I, Cardiology & Vascular Medicine, Aachen, Germany; ^2^Cardiovascular Research Institute Maastricht (CARIM), Physiology, Maastricht, the Netherlands;^3^Department of Electronics, Information and Bioengineering, Politecnico di Milano, Italy; ^4^Center for Computational Modeling in Cardiology, Switzerland; ^5^Department of Cardiology, Cardiovascular Research Institute Maastricht (CARIM), Maastricht University Medical Center, Maastricht, the Netherlands; ^6^Ziekenhuis Oost Limburg, Genk, Belgium; ^7^Department of Cardiology, Radboud University Medical Center, Nijmegen, the Netherlands; ^8^Instituto Cardiocentro Ticino, Lugano, Switzerland*

* These authors have contributed equally to the work and share first authorship

**Table 1) P-wave features**

|  |  | **Total** | **paroxysmal** | **persistent** | ***P=*** |
| --- | --- | --- | --- | --- | --- |
|  |  | Mean±SD | Mean±SD | Mean±SD | *P* |
| N= |  | 164 | 121 | 43 |  |
|  |  |  |  |  |  |
|  |  |  |  |  |  |
| **12-lead ECG** |  |  |  |  |  |
| P-wave duration [ms] | | 117±17 | 118±16 | 113±18 | 0.06 |
| PQ time [ms] |  | 181±28 | 180±28 | 184±27 | 0.271 |
| QRS width [ms] | | 99±24 | 97±22 | 102±27 | 0.352 |
| QT time [ms] |  | 421±51 | 424±50 | 415±52 | 0.117 |
| **RR interval [ms]** | | **911±219** | **944±215** | **862±216** | **0.001** |
| **Heart rate [/min]** | | **71±21** | **68±21** | **74±21** | **0.001** |
|  | |  |  |  |  |
|  | |  |  |  |  |
|  | |  |  |  |  |
| **Signal averaged P-wave** | |  |  |  |  |
| Global P-wave duration [ms] | | 141±21 | 139±18 | 148±27 | 0.06 |
| PQ time [ms] |  | 183±27 | 182±27 | 186±28 | 0.361 |
| Terminal force [mV*ms] | | 2.4±2.7 | 2.3±1.4 | 2.7±4.6 | 0.225 |
| Area [mV*ms] | A1 | 4.8±3.77 | 4.72±2.21 | 5.02±6.41 | 0.117 |
|  | A2 | 3.13±1.43 | 3.15±1.19 | 3.04±1.99 | 0.243 |
|  | A3 | 1.91±0.94 | 1.93±0.94 | 1.84±0.94 | 0.472 |
|  | I | 4.8±2.7 | 4.86±2.06 | 4.64±4.03 | 0.09 |
|  | II | 8.07±4.37 | 8.07±2.92 | 8.08±7.05 | 0.091 |
|  | III | 4.69±6.05 | 4.23±1.98 | 5.96±11.34 | 0.948 |
|  | V1 | 4±2.79 | 3.82±1.61 | 4.53±4.72 | 0.833 |
|  | **V2** | **3.62±4.19** | **3.19±1.33** | **4.82±7.82** | **0.024** |
|  | V3 | 4.62±3.03 | 4.42±1.42 | 5.17±5.42 | 0.987 |
|  | V4 | 4.74±2.64 | 4.65±1.56 | 4.98±4.48 | 0.347 |
|  | V5 | 4.54±2.99 | 4.43±1.5 | 4.85±5.3 | 0.244 |
|  | V6 | 4.28±3.73 | 4.14±1.5 | 4.68±6.86 | 0.083 |
|  | aVF | 6.07±5.12 | 5.85±2.44 | 6.71±9.18 | 0.241 |
|  | aVL | 2.63±4.08 | 2.31±1.2 | 3.54±7.7 | 0.279 |
|  | **aVR** | **6.21±2.19** | **6.4±2.19** | **5.66±2.14** | **0.05** |
|  |  |  |  |  |  |
| Amplitude [mV] | A1 | 0.089±0.05 | 0.089±0.036 | 0.091±0.079 | 0.207 |
|  | **A2** | **0.058±0.03** | **0.058±0.019** | **0.057±0.05** | **0.037** |
|  | A3 | 0.052±0.039 | 0.05±0.018 | 0.056±0.071 | 0.334 |
|  | I | 0.087±0.043 | 0.089±0.033 | 0.083±0.065 | 0.029 |
|  | II | 0.145±0.052 | 0.144±0.044 | 0.146±0.072 | 0.334 |
|  | III | 0.104±0.069 | 0.099±0.035 | 0.12±0.121 | 0.541 |
|  | V1 | 0.117±0.08 | 0.112±0.043 | 0.131±0.138 | 0.91 |
|  | V2 | 0.104±0.077 | 0.097±0.038 | 0.126±0.136 | 0.103 |
|  | V3 | 0.112±0.072 | 0.105±0.03 | 0.131±0.131 | 0.433 |
|  | V4 | 0.094±0.032 | 0.092±0.024 | 0.099±0.048 | 0.844 |
|  | V5 | 0.081±0.032 | 0.079±0.022 | 0.084±0.051 | 0.579 |
|  | V6 | 0.073±0.045 | 0.072±0.022 | 0.077±0.08 | 0.092 |
|  | aVF | 0.116±0.056 | 0.113±0.038 | 0.125±0.089 | 0.693 |
|  | aVL | 0.065±0.052 | 0.063±0.025 | 0.072±0.092 | 0.707 |
|  | aVR | 0.109±0.034 | 0.111±0.032 | 0.102±0.037 | 0.063 |
|  |  |  |  |  |  |
| Shannon Entropy [au] | A1 | 3.06±0.12 | 3.07±0.11 | 3.04±0.16 | 0.408 |
|  | A2 | 3.1±0.16 | 3.11±0.12 | 3.06±0.24 | 0.433 |
|  | A3 | 2.99±0.19 | 3.01±0.14 | 2.96±0.29 | 0.486 |
|  | I | 3.09±0.11 | 3.09±0.11 | 3.1±0.11 | 0.761 |
|  | II | 3.12±0.11 | 3.13±0.1 | 3.1±0.12 | 0.063 |
|  | III | 3.09±0.13 | 3.09±0.13 | 3.07±0.13 | 0.253 |
|  | **V1** | **3.03±0.16** | **3.04±0.13** | **2.97±0.23** | **0.033** |
|  | V2 | 3±0.15 | 3.01±0.14 | 2.96±0.18 | 0.099 |
|  | V3 | 3.06±0.16 | 3.08±0.13 | 3.02±0.21 | 0.055 |
|  | V4 | 3.13±0.11 | 3.14±0.1 | 3.09±0.14 | 0.051 |
|  | V5 | 3.14±0.1 | 3.14±0.1 | 3.12±0.1 | 0.436 |
|  | V6 | 3.13±0.1 | 3.14±0.1 | 3.11±0.11 | 0.218 |
|  | **aVF** | **3.11±0.11** | **3.12±0.11** | **3.08±0.12** | **0.01** |
|  | **aVL** | **3.01±0.13** | **2.99±0.14** | **3.05±0.12** | **0.033** |
|  | aVR | 3.11±0.13 | 3.12±0.1 | 3.1±0.18 | 0.99 |
|  |  |  |  |  |  |
| Sample Entropy [au] | A1 | 0.267±0.097 | 0.262±0.096 | 0.28±0.099 | 0.193 |
|  | **A2** | **0.231±0.078** | **0.225±0.078** | **0.249±0.076** | **0.035** |
|  | A3 | 0.248±0.119 | 0.254±0.131 | 0.232±0.072 | 0.886 |
|  | **I** | **0.225±0.089** | **0.213±0.072** | **0.259±0.119** | **0.006** |
|  | II | 0.218±0.056 | 0.218±0.058 | 0.22±0.051 | 0.713 |
|  | III | 0.288±0.113 | 0.293±0.116 | 0.272±0.101 | 0.202 |
|  | **V1** | **0.221±0.044** | **0.225±0.042** | **0.211±0.046** | **0.024** |
|  | **V2** | **0.243±0.087** | **0.253±0.092** | **0.215±0.065** | **0.009** |
|  | V3 | 0.222±0.06 | 0.225±0.059 | 0.214±0.062 | 0.2 |
|  | V4 | 0.236±0.063 | 0.237±0.062 | 0.232±0.066 | 0.579 |
|  | V5 | 0.234±0.061 | 0.232±0.06 | 0.24±0.062 | 0.571 |
|  | V6 | 0.224±0.055 | 0.22±0.053 | 0.238±0.057 | 0.084 |
|  | aVF | 0.24±0.071 | 0.242±0.071 | 0.235±0.07 | 0.615 |
|  | aVL | 0.28±0.104 | 0.28±0.107 | 0.281±0.099 | 0.786 |
|  | **aVR** | **0.218±0.055** | **0.212±0.051** | **0.237±0.063** | **0.011** |
|  |  |  |  |  |  |
| Complexity [N] | **A1** | **2.7±1.7** | **2.4±1.5** | **3.3±2.1** | **0.008** |
|  | A2 | 2.1±1.5 | 2±1.1 | 2.6±2.3 | 0.129 |
|  | A3 | 2.6±1.5 | 2.5±1.3 | 2.7±2.1 | 0.798 |
|  | I | 2.1±1.7 | 2±1.2 | 2.4±2.5 | 0.536 |
|  | **II** | **2±1.3** | **1.9±1.2** | **2.4±1.6** | **0.025** |
|  | III | 3±1.5 | 2.8±1.4 | 3.4±1.8 | 0.06 |
|  | **V1** | **2.2±0.9** | **2.1±0.7** | **2.4±1.4** | **0.037** |
|  | V2 | 2.4±1.4 | 2.4±1.2 | 2.6±1.9 | 0.904 |
|  | V3 | 2.2±1.4 | 2.1±1.2 | 2.2±1.9 | 0.689 |
|  | V4 | 2.1±1.3 | 2±1.2 | 2.4±1.5 | 0.112 |
|  | **V5** | **2.1±1.3** | **2±1.2** | **2.6±1.6** | **0.024** |
|  | **V6** | **2±1.3** | **1.8±1.1** | **2.4±1.7** | **0.009** |
|  | aVF | 2.3±1.4 | 2.2±1.4 | 2.6±1.5 | 0.197 |
|  | aVL | 3.2±1.6 | 3.1±1.5 | 3.5±1.8 | 0.183 |
|  | **aVR** | **1.9±1.3** | **1.7±1** | **2.4±1.9** | **0.014** |
|  |  |  |  |  |  |
| **Spatio-temporal P-wave variability** | | |  |  |  |
| *N*= |  | 159 | 119 | 40 |  |
|  |  |  |  |  |  |
| Euclidean Distance | **A1** | **(4.10±2.10) x 10-1** | **(3.91±2.03) x 10^-1** | **(4.67±2.21) x 10^-1** | **0.015** |
|  | **A2** | **(4.71±2.22) x 10^-1** | **(4.47±2.06) x 10^-1** | **(5.40±2.55) x 10^-1** | **0.001** |
|  | A3 | (5.69±2.33) x 10^-1 | (5.59±2.34) x 10^-1 | (5.98±2.28) x 10^-1 | 0.086 |
|  | **I** | **(4.83±2.63) x 10^-1** | **(4.52±2.41) x 10^-1** | **(5.73±3.07) x 10^-1** | **0.001** |
|  | **II** | **(2.99±1.32) x 10^-1** | **(2.86±1.18) x 10^-1** | **(3.37±1.63) x 10^-1** | **0.009** |
|  | III | (4.64±1.91) x 10^-1 | (4.62±1.98) x 10^-1 | (4.69±1.72) x 10^-1 | 0.626 |
|  | V1 | (2.88±1.31) x 10^-1 | (2.87±1.32) x 10^-1 | (2.90±1.31) x 10^-1 | 0.660 |
|  | V2 | (3.04±1.42) x 10^-1 | (3.13±1.51) x 10^-1 | (2.77±1.08) x 10^-1 | 0.171 |
|  | V3 | (2.52±0.89) x 10^-1 | (2.50±0.86) x 10^-1 | (2.57±0.99) x 10^-1 | 0.925 |
|  | V4 | (3.17±1.25) x 10^-1 | (3.12±1.18) x 10^-1 | (3.31±1.43) x 10^-1 | 0.389 |
|  | V5 | (3.66±1.38) x 10^-1 | (3.59±1.36) x 10^-1 | (3.86±1.41) x 10^-1 | 0.218 |
|  | **V6** | **(4.11±1.71) x 10^-1** | **(3.87±1.48) x 10^-1** | **(4.80±2.12) x 10^-1** | **0.002** |
|  | **aVR** | **(3.30±1.64) x 10^-1** | **(3.09±1.44) x 10^-1** | **(3.89±2.03) x 10^-1** | **0.001** |
|  | aVL | (6.12±2.50) x 10^-1 | (6.05±2.51) x 10^-1 | (6.32±2.49) x 10^-1 | 0.356 |
|  | aVF | (3.49±1.41) x 10^-1 | (3.42±1.37) x 10^-1 | (3.69±1.51) x 10^-1 | 0.170 |
|  | **PC1** | **(2.37±1.14) x 10^-1** | **(2.28±1.09) x 10^-1** | **(2.62±1.26) x 10^-1** | **0.009** |
|  | PC2 | (2.73±1.16) x 10^-1 | (2.75±1.20) x 10^-1 | (2.68±1.03) x 10^-1 | 0.989 |
|  | PC3 | (5.32±2.59) x 10^-1 | (5.28±2.58) x 10^-1 | (5.44±2.65) x 10^-1 | 0.354 |
|  |  |  |  |  |  |
| Similarity Index | **A1** | **(9.08±0.93) x 10^-1** | **(9.19±0.81) x 10^-1** | **(8.76±1.16) x 10^-1** | **0.011** |
|  | **A2** | **(8.82±1.20) x 10^-1** | **(9.01±0.89) x 10^-1** | **(8.28±1.71) x 10^-1** | **<0.001** |
|  | A3 | (8.22±1.47) x 10^-1 | (8.29±1.45) x 10^-1 | (8.04±1.52) x 10^-1 | 0.147 |
|  | **I** | **(8.63±1.48) x 10^-1** | **(8.86±1.20) x 10^-1** | **(7.96±1.96) x 10^-1** | **0.001** |
|  | **II** | **(9.57±0.35) x 10^-1** | **(9.61±0.30) x 10^-1** | **(9.45±0.45) x 10^-1** | **0.008** |
|  | III | (8.95±0.80) x 10^-1 | (8.96±0.81) x 10^-1 | (8.91±0.77) x 10^-1 | 0.981 |
|  | V1 | (9.60±0.33) x 10^-1 | (9.60±0.34) x 10^-1 | (9.62±0.29) x 10^-1 | 0.726 |
|  | V2 | (9.62±0.28) x 10^-1 | (9.60±0.30) x 10^-1 | (9.67±0.23) x 10^-1 | 0.190 |
|  | V3 | (9.71±0.18) x 10^-1 | (9.72±0.17) x 10^-1 | (9.69±0.21) x 10^-1 | 0.839 |
|  | V4 | (9.50±0.37) x 10^-1 | (9.50±0.38) x 10^-1 | (9.48±0.36) x 10^-1 | 0.991 |
|  | V5 | (9.31±0.52) x 10^-1 | (9.34±0.50) x 10^-1 | (9.23±0.58) x 10^-1 | 0.195 |
|  | **V6** | **(9.18±0.64) x 10^-1** | **(9.29±0.49) x 10^-1** | **(8.87±0.89) x 10^-1** | **0.001** |
|  | **aVR** | **(9.44±0.55) x 10^-1** | **(9.52±0.45) x 10^-1** | **(9.21±0.72) x 10^-1** | **0.002** |
|  | aVL | (7.98±1.63) x 10^-1 | (8.04±1.63) x 10^-1 | (7.79±1.64) x 10^-1 | 0.350 |
|  | aVF | (9.43±0.40) x 10^-1 | (9.46±0.38) x 10^-1 | (9.35±0.43) x 10^-1 | 0.250 |
|  | **PC1** | **(9.72±0.27) x 10^-1** | **(9.76±0.22) x 10^-1** | **(9.61±0.37) x 10^-1** | **0.001** |
|  | PC2 | (9.63±0.30) x 10^-1 | (9.62±0.32) x 10^-1 | (9.66±0.23) x 10^-1 | 0.966 |
|  | PC3 | (8.52±1.36) x 10^-1 | (8.49±1.42) x 10^-1 | (8.60±1.18) x 10^-1 | 0.929 |
|  |  |  |  |  |  |
| Spatial similarity [%] |  | **94.40 ± 2.70** | **94.75 ± 2.30** | **93.33 ± 3.46** | **0.001** |

**Table 2) f-wave features**

|  |  | **Total** | **paroxysmal** | **persistent** | ***P=*** |
| --- | --- | --- | --- | --- | --- |
|  |  | Mean±SD | Mean±SD | Mean±SD |  |
| N= |  | 83 | 31 | 52 |  |
|  |  |  |  |  |  |
| Dominant frequency [Hz] | **A1** | **6.5±0.91** | **6.23±0.73** | **6.65±0.98** | **0.047** |
|  | A2 | 6.51±0.82 | 6.41±0.75 | 6.56±0.86 | 0.424 |
|  | A3 | 6.49±1.09 | 6.44±1.24 | 6.52±1 | 0.275 |
|  | I | 6.55±0.83 | 6.48±0.76 | 6.59±0.87 | 0.494 |
|  | II | 6.34±0.83 | 6.29±0.76 | 6.36±0.88 | 0.729 |
|  | III | 6.33±0.9 | 6.11±0.91 | 6.45±0.88 | 0.131 |
|  | V1 | 6.58±0.99 | 6.46±0.89 | 6.65±1.04 | 0.507 |
|  | V2 | 6.66±1.08 | 6.67±1.21 | 6.65±1.02 | 0.492 |
|  | V3 | 6.44±0.89 | 6.45±0.77 | 6.44±0.96 | 0.94 |
|  | V4 | 6.46±1.09 | 6.56±1.23 | 6.41±1.02 | 0.961 |
|  | V5 | 6.49±1.01 | 6.5±1.18 | 6.49±0.91 | 0.708 |
|  | V6 | 6.43±0.83 | 6.27±0.72 | 6.52±0.87 | 0.144 |
|  | aVF | 6.36±0.78 | 6.21±0.64 | 6.44±0.84 | 0.209 |
|  | aVL | 6.51±1.02 | 6.44±1.24 | 6.55±0.89 | 0.214 |
|  | aVR | 6.43±0.92 | 6.41±1 | 6.43±0.88 | 0.787 |
|  |  |  |  |  |  |
| Organization index | A1 | 0.35±0.14 | 0.34±0.13 | 0.36±0.14 | 0.631 |
|  | A2 | 0.25±0.08 | 0.23±0.09 | 0.27±0.08 | 0.097 |
|  | A3 | 0.27±0.12 | 0.27±0.12 | 0.27±0.11 | 0.858 |
|  | I | 0.24±0.12 | 0.22±0.11 | 0.25±0.12 | 0.216 |
|  | II | 0.33±0.12 | 0.31±0.12 | 0.34±0.12 | 0.396 |
|  | III | 0.35±0.13 | 0.33±0.14 | 0.36±0.13 | 0.28 |
|  | V1 | 0.46±0.13 | 0.43±0.13 | 0.47±0.12 | 0.117 |
|  | V2 | 0.37±0.11 | 0.34±0.11 | 0.38±0.1 | 0.16 |
|  | V3 | 0.35±0.11 | 0.35±0.13 | 0.34±0.1 | 0.9 |
|  | V4 | 0.31±0.1 | 0.31±0.1 | 0.31±0.1 | 0.973 |
|  | V5 | 0.3±0.09 | 0.3±0.1 | 0.3±0.09 | 0.789 |
|  | V6 | 0.28±0.1 | 0.29±0.1 | 0.28±0.1 | 0.854 |
|  | aVF | 0.36±0.13 | 0.35±0.14 | 0.36±0.12 | 0.684 |
|  | aVL | 0.3±0.12 | 0.29±0.13 | 0.31±0.12 | 0.329 |
|  | aVR | 0.27±0.11 | 0.27±0.11 | 0.28±0.12 | 0.724 |
|  |  |  |  |  |  |
| Regularity index | A1 | 0.31±0.09 | 0.31±0.09 | 0.3±0.09 | 0.762 |
|  | A2 | 0.24±0.05 | 0.23±0.05 | 0.25±0.05 | 0.139 |
|  | A3 | 0.27±0.07 | 0.26±0.08 | 0.27±0.07 | 0.783 |
|  | I | 0.25±0.07 | 0.24±0.05 | 0.26±0.08 | 0.204 |
|  | II | 0.3±0.08 | 0.29±0.09 | 0.3±0.08 | 0.624 |
|  | III | 0.31±0.09 | 0.3±0.09 | 0.31±0.09 | 0.67 |
|  | V1 | 0.36±0.1 | 0.35±0.1 | 0.37±0.1 | 0.533 |
|  | V2 | 0.29±0.08 | 0.29±0.1 | 0.29±0.08 | 0.94 |
|  | V3 | 0.28±0.08 | 0.28±0.1 | 0.28±0.07 | 0.805 |
|  | V4 | 0.27±0.08 | 0.26±0.09 | 0.27±0.07 | 0.604 |
|  | V5 | 0.26±0.07 | 0.26±0.08 | 0.26±0.06 | 0.776 |
|  | V6 | 0.27±0.07 | 0.27±0.07 | 0.26±0.07 | 0.813 |
|  | aVF | 0.31±0.09 | 0.31±0.09 | 0.31±0.09 | 0.615 |
|  | aVL | 0.28±0.08 | 0.27±0.08 | 0.29±0.08 | 0.392 |
|  | aVR | 0.27±0.07 | 0.26±0.07 | 0.27±0.07 | 0.813 |
|  |  |  |  |  |  |
| Spectral entropy | A1 | 8.9±0.91 | 8.91±1.03 | 8.89±0.84 | 0.9 |
|  | A2 | 9.35±0.9 | 9.24±0.99 | 9.41±0.85 | 0.538 |
|  | A3 | 9.56±0.98 | 9.45±1.19 | 9.63±0.85 | 0.438 |
|  | I | 9.69±0.92 | 9.69±0.91 | 9.69±0.93 | 0.87 |
|  | II | 9.05±0.98 | 9.08±1.07 | 9.03±0.93 | 0.843 |
|  | III | 8.91±0.99 | 8.86±1.12 | 8.94±0.91 | 0.713 |
|  | V1 | 8.02±0.93 | 8.06±1.03 | 8±0.88 | 0.32 |
|  | V2 | 8.49±0.82 | 8.5±0.96 | 8.48±0.74 | 0.532 |
|  | V3 | 8.68±0.83 | 8.61±1.02 | 8.73±0.7 | 0.698 |
|  | V4 | 9±0.86 | 8.88±0.99 | 9.07±0.78 | 0.362 |
|  | V5 | 9.1±0.86 | 8.97±0.96 | 9.18±0.8 | 0.378 |
|  | V6 | 9.24±0.88 | 9.08±0.96 | 9.33±0.83 | 0.225 |
|  | aVF | 8.85±0.98 | 8.81±1.13 | 8.87±0.89 | 0.795 |
|  | aVL | 9.33±0.95 | 9.28±1.01 | 9.35±0.92 | 0.732 |
|  | aVR | 9.45±0.93 | 9.4±0.95 | 9.47±0.93 | 0.748 |
|  |  |  |  |  |  |
| f-wave amplitude [µV] | A1 | 74.13±22.5 | 78.19±23.55 | 71.83±21.78 | 0.218 |
|  | A2 | 48.55±15.2 | 52.5±18.33 | 46.31±12.76 | 0.118 |
|  | **A3** | **49.82±16.33** | **56.18±18.88** | **46.22±13.61** | **0.011** |
|  | I | 75.44±31.79 | 81.79±35.27 | 71.78±29.33 | 0.218 |
|  | II | 90.31±32.1 | 99.36±35.19 | 85.36±29.45 | 0.083 |
|  | III | 91.86±33.13 | 100.93±36.4 | 86.72±30.29 | 0.062 |
|  | V1 | 103.06±42.81 | 107.4±35.25 | 100.6±46.69 | 0.092 |
|  | V2 | 87.11±37.13 | 90.92±31.42 | 84.95±40.13 | 0.483 |
|  | V3 | 76.9±28.77 | 82.97±28.74 | 73.46±28.49 | 0.144 |
|  | **V4** | **67.98±21.2** | **74.22±21.55** | **64.56±20.4** | **0.036** |
|  | **V5** | **59.15±18.04** | **64.48±18.07** | **56.14±17.48** | **0.03** |
|  | **V6** | **52.39±15.37** | **57.79±16.97** | **49.34±13.62** | **0.014** |
|  | **aVF** | **83.05±28.47** | **91.99±31.73** | **77.99±25.38** | **0.043** |
|  | aVL | 73.26±30.14 | 80.34±32.81 | 69.25±28.05 | 0.083 |
|  | aVR | 71.47±28.79 | 77.66±33.85 | 67.96±25.17 | 0.232 |

**Table 3) Best Correlation of clinical and echocardiographic parameter with P-wave and f-wave features; LA: Left atrial; RA: Right atrial; LVEF: Left ventricular ejection fraction**

| **Parameter** | **Parameter & Lead** | ***rho*** | ***P*** | ***N=*** | **AF-Type** | ***rho*** | ***P*** | ***N=*** |
| --- | --- | --- | --- | --- | --- | --- | --- | --- |
| **Signal-averaged P-wave**  CHA_2_DS_2_ VASc | Complexity I | **-0.266** | **0.001** | **164** | **paroxysmal** | **-0.260** | **0.004** | 121 |
|  |  |  |  |  | persistent | -0.272 | 0.077 | 43 |
| LVEF [%] | Area III | **-0.223** | **0.007** | **147** | **paroxysmal** | **-0.189** | **0.050** | 108 |
|  |  |  |  |  | **persistent** | **-0.367** | **0.022** | 39 |
| LA diameter [mm] | Global P-wave duration | **0.317** | **<0.001** | **132** | **paroxysmal** | **0.249** | **0.016** | 94 |
|  |  |  |  |  | **persistent** | **0.493** | **0.002** | 38 |
| LA volume [ml] | Global P-wave duration | **0.245** | **0.006** | **125** | **paroxysmal** | **0.236** | **0.025** | 90 |
|  |  |  |  |  | persistent | 0.089 | 0.610 | 35 |
| RA volume [ml] | Shannon Entropy A1 | **0.262** | **0.007** | **105** | **paroxysmal** | **0.259** | **0.027** | 73 |
|  |  |  |  |  | persistent | 0.307 | 0.088 | 32 |
| **P-wave variability**  CHA_2_DS_2_ VASc | Euclidean Distance V4 | **0.379** | **<0.001** | **159** | **paroxysmal** | **0.409** | **<0.001** | 119 |
|  |  |  |  |  | persistent | 0.280 | 0.080 | 40 |
| LA diameter [mm] | Similarity Index aVF | **-0.220** | **0.016** | **123** | **paroxysmal** | **-0.231** | **0.032** | 88 |
|  |  |  |  |  | persistent | -0.130 | 0.463 | 35 |
| LA volume [ml] | Similarity Index V6 | -0.197 | 0.035 | 120 | paroxysmal | -0.054 | 0.629 | 88 |
|  |  |  |  |  | persistent | -0.204 | 0.271 | 32 |
|  |  |  |  |  |  |  |  |  |
| **f-wave** |  |  |  |  |  |  |  |  |
| CHA_2_DS_2_ VASc | Organization Index I | -0.333 | 0.002 | 82 | paroxysmal | -0.357 | 0.053 | 30 |
|  |  |  |  |  | **persistent** | **-0.275** | **0.049** | 52 |
| LVEF [%] | f-wave amplitude aVF | 0.338 | 0.003 | 73 | paroxysmal | 0.178 | 0.406 | 24 |
|  |  |  |  |  | **persistent** | **0.342** | **0.016** | 49 |
| LA diameter [mm] | f-wave amplitude A3 | **-0.389** | **0.002** | **63** | **paroxysmal** | **-0.486** | **0.03** | 20 |
|  |  |  |  |  | **persistent** | **-0.353** | **0.02** | 43 |
| LA volume [ml] | f-wave amplitude III | -0.286 | 0.033 | 56 | paroxysmal | -0.084 | 0.757 | 16 |
|  |  |  |  |  | persistent | -0.307 | 0.054 | 40 |
| RA volume [ml] | f-wave amplitude aVL | -0.343 | 0.021 | 45 | paroxysmal | -0.051 | 0.863 | 14 |
|  |  |  |  |  | paroxysmal | -0.482 | 0.006 | 31 |

**Table 4) Classification of AF type based on P-wave and f-wave parameters in patients undergoing their first ablation procedure**

|  | | **AUC** | **Sensitivity** | **Specificity** |
| --- | --- | --- | --- | --- |
| **Parameter** | **Lead** | Median (IQR) |  |  |
| *P-wave parameters (n = 97 paroxysmal AF, 25 persistent AF)* | | | | |
| Entropy | A3 | 0.63 (0.57-0.71) | 0.53 | 0.63 |
| Area | V2 | 0.62 (0.55-0.69) | 0.68 | 0.51 |
| Similarity Index | A2 | 0.59 (0.51-0.71) | 0.63 | 0.53 |
| Model # |  | 0.83 (0.76-0.87) | 0.80 | 0.68 |
| *f-wave parameters (n = 14 paroxysmal AF, 39 persistent AF* | | | | |
| Amplitude | A3 | 0.78 (0.71-0.92) | 0.79 | 0.76 |
| Model $ |  | 0.80 (0.71-0.92) | 0.85 | 0.68 |

**Indices shown represent the highest AUC for a single indice in our P- (Routine 12-lead ECG: Area V2; Signal-averaged P-wave: Entropy A3; Spatio-temporal anaylsis: Similarity index A2) and f-wave (dominant frequency A1) analysis.**

**AUC: Area under the receiver operating characteristic curve; IQR: interquartile range**

**# P-wave model includes: Amplitude I, Shannon entropy A2/I/aVL, sample entropy V2, complexity III, similarity index V6/A2/PCA2**

**$ f-wave model includes: f-wave amplitude A3, and organization index V2**
